# Isolation of Single-Stranded DNA Aptamers That Distinguish Influenza Virus Hemagglutinin Subtype H1 from H5

**DOI:** 10.1371/journal.pone.0125060

**Published:** 2015-04-22

**Authors:** Hye-Min Woo, Jin-Moo Lee, Sanggyu Yim, Yong-Joo Jeong

**Affiliations:** Department of Bio and Nanochemistry, Kookmin University, Seoul 136–702, Republic of Korea; Consiglio Nazionale delle Ricerche (CNR), ITALY

## Abstract

Surface protein hemagglutinin (HA) mediates the binding of influenza virus to host cell receptors containing sialic acid, facilitating the entry of the virus into host cells. Therefore, the HA protein is regarded as a suitable target for the development of influenza virus detection devices. In this study, we isolated single-stranded DNA (ssDNA) aptamers binding to the HA1 subunit of subtype H1 (H1-HA1), but not to the HA1 subunit of subtype H5 (H5-HA1), using a counter-systematic evolution of ligands by exponential enrichment (counter-SELEX) procedure. Enzyme-linked immunosorbent assay and surface plasmon resonance studies showed that the selected aptamers bind tightly to H1-HA1 with dissociation constants in the nanomolar range. Western blot analysis demonstrated that the aptamers were binding to H1-HA1 in a concentration-dependent manner, yet were not binding to H5-HA1. Interestingly, the selected aptamers contained G-rich sequences in the central random nucleotides region. Further biophysical analysis showed that the G-rich sequences formed a G-quadruplex structure, which is a distinctive structure compared to the starting ssDNA library. Using flow cytometry analysis, we found that the aptamers did not bind to the receptor-binding site of H1-HA1. These results indicate that the selected aptamers that distinguish H1-HA1 from H5-HA1 can be developed as unique probes for the detection of the H1 subtype of influenza virus.

## Introduction

Influenza viruses are responsible for serious respiratory diseases and are deemed to be one of the biggest threats to human health. Belonging to the family *Orthomyxoviridae*, influenza virus is an enveloped virus with single-stranded negative-sense RNA consisting of eight segments [1]. Its two major surface glycoproteins, hemagglutinin (HA) and neuraminidase (NA) [2], are highly expressed during viral infection and serve as targets for immune detection. Thus far, 18 HA (H1–H18) and 11 NA (N1–N11) have been identified [3], and influenza is classified on the basis of the subtypes of HA and NA proteins. Subtype H5 is known as highly pathogenic in poultry [4], while H1N1 is known as a seasonal strain and caused pandemics such as the 1918 Spanish flu and the 2009 swine flu [5].

Hemagglutinin is the most abundant protein on the viral surface and displays epitopes that trigger neutralizing antibody production. It is initially synthesized as a precursor HA0 protein, which is proteolytically cleaved into HA1 and HA2 subunits [6]. In the mature HA homotrimer of approximately 220 kDa, HA2 is embedded in the viral membrane, whereas HA1 mediates the binding to the host cell surface [7]. Infection is initiated by binding of HA1 to sialic acid on the membranes of respiratory epithelial cells [8], which makes HA1 a primary target for the development of antiviral agents and diagnostics [9,10]. Thus far, diagnostic methods for the detection of influenza virus infections were mainly based on reverse transcription polymerase chain reaction (RT-PCR) [11,12], enzyme-linked immunosorbent assay (ELISA) [13], and real-time PCR (qPCR) [14].

Aptamers are nucleic acids binding to specific target molecules with high affinities, produced by systematic evolution of ligands by exponential enrichment (SELEX) [15]. Thus far, aptamers have been developed for a wide range of purposes such as biosensors, medical diagnosis, and therapy [16,17]. In order to improve aptamer production, enhanced versions of SELEX were established, such as counter-SELEX, toggle-SELEX, cell-SELEX, and others [18]. Using counter-SELEX methods, the Polisky group created an RNA aptamer that could distinguish between theophylline and caffeine, differing by only one methyl group [19], while the Famulok group made aptamers discriminating between cytohesin-1 and cytohesin-2 [20]. As these and other previous studies indicated, aptamers generated by counter-SELEX can discern minute differences between targets of closely related structures [21,22]. Based on this sensitivity, it can be assumed that aptamers selected by counter-SELEX might be ideal diagnostic tools for differentiating between HA subtypes.

In the current study, we used counter-SELEX to isolate ssDNA aptamers that distinguish between H1-HA1 protein and H5-HA1 protein. The selected ssDNA aptamers showed high binding affinities to H1-HA1 *in vitro*, yet did not block the sialic acid-binding region of HA1. Based on the power of discrimination between such closely related structures, it is suggested that aptamers are the probes of choice for the detection of influenza subtypes.

## Materials and Methods

### PCR and ssDNA generation

A library of DNA molecules composed of two primer regions and a 45-nucleotide random region was prepared, and ssDNAs were generated as previously described, with slight modifications [23]. All synthetic DNAs were purchased from Integrated DNA Technologies (IDT, Coralville, IA,). Asymmetric PCR reactions (100 μL) were set up with a molar primer ratio of 100:1 (forward primer:reverse primer), and contained 0.2 mM dNTPs, 1 μM forward primer, 10 nM 5′-phosphorylated reverse primer, 10 nM template, and 2.5 U Taq DNA polymerase. The mixture was thermally cycled 20 times at 95°C for 1 min, 52°C for 30 s, and 72°C for 45 s, followed by a 7 min extension step at 72°C. After PCR, purified dsDNA was incubated with 25 U of lambda exonuclease (NEB, Frankfurt am Main, Germany) at 37°C for 3 h. The reaction was terminated by incubation at 75°C for 10 min. The products of the digested strand were analyzed by electrophoresis in a 10% polyacrylamide/8 M urea TBE gel, and ssDNA was purified from the gel for the next round of selection.

### Counter-SELEX procedure

*In vitro* selection of ssDNA aptamers that distinguish between H1-HA1 and H5-HA1 was performed as previously described, with slight modifications [23,24]. First, 2 μg of the opponent protein (GST-H5-HA1) was incubated with 100 μL of glutathione agarose beads in 100 μL of binding buffer (50 mM Tris/HCl; pH 8.0, 150 mM NaCl, 1.5 mM MgCl_2_, 2 mM DTT, and 1% [w/v] BSA) for 30 min at room temperature with occasional shaking. The synthetic ssDNA library was denatured by heating at 95°C for 10 min and immediately annealed on ice for 10 min. Second, 2 μg of the DNA library was incubated with the opponent protein bound to glutathione agarose beads for 30 min at room temperature. Bead/opponent protein-bound DNA was precipitated and discarded.

The pre-cleared supernatant was collected and incubated with 2 μg target protein (GST-H1-HA1) in 100 μL binding buffer for 30 min at room temperature. Glutathione agarose beads (100 μL) were added, the reaction mixture was incubated for another 30 min, centrifuged to remove unbound DNA molecules, and the pellets were washed five times with 500 μL binding buffer. The GST-H1-HA1—ssDNA pool complexes were dissociated from the glutathione agarose beads with elution buffer (binding buffer plus 10 mM glutathione). ssDNA pool bound to GST-H1-HA1 were recovered from the supernatant by phenol—chloroform extraction and ethanol precipitation. Using this procedure, a total of 14 sequential selection rounds were performed, applying more stringent conditions from round 8 onwards, by increasing the opponent protein concentration (4 μg: rounds 8–9, 8 μg: rounds 10–11, 16 μg: rounds 12–13, and 32 μg: round 14) and reducing the target protein concentration (1 μg: rounds 8–9, 0.5 μg: rounds 10–11, 0.25 μg: rounds 12–13, and 0.125 μg: round 14). After the 14^th^ round, ssDNA was amplified by PCR, cloned into a linearized pGEM-T vector (Promega, Madison, WI), and DNA sequences were determined as previously described [23]. Sequence alignment of selected aptamers was performed by ClustalW2 [25], and secondary structures were predicted using Mfold [26].

### HA1 protein-ssDNA aptamer binding analysis by ELISA

Aptamers were 5′-biotinylated by asymmetric PCR using the forward primer 5′-Biotin-GCAATGTACGGTACTTCC-3′ followed by lambda exonuclease digestion, as previously described [27,28]. The 5′-biotinylated ssDNA aptamers (100 nM) were heated at 90°C for 10 min, immediately placed on ice, added to the wells of a streptavidin-coated plate (Pierce Biotechnology, Rockford, IL), and incubated for 1 h at room temperature while shaking at 100 rpm. The wells were washed four times with PBST (0.1% Tween 20 in PBS; pH 7.4), blocked with 5% BSA in PBST at room temperature for 1 h, re-washed four times, and incubated with various concentrations of purified GST-H1-HA1 in PBS at room temperature for 1 h. After washing four times with PBST, incubation with GST antibody-conjugated horseradish peroxidase (HRP; 1:1,000 in PBST, Santa Cruz Biotechnology, Dallas, TX) at room temperature for 1 h, and four additional washes, bound GST-tagged HA1 protein was detected by adding 3,3′,5,5′-Tetramethylbenzidine (TMB) solution (Merck, Darmstadt, Germany) and terminating with 0.5 N H_2_SO_4_. The absorbance of each well was measured at 450 nm by using a TRIAD microplate reader (Dynex Technologies, Chantilly, VA). GST-H5-HA1 and GST served as negative controls.

### Affinity (K_D_) determination by surface plasmon resonance

For binding affinity measurements, the surface plasmon resonance (SPR) technology-based ProteON XPR36 (Bio-Rad, Hercules, CA) was used with PBS (pH 7.4) at 25°C. Biotinylated ssDNA aptamers (0.25 μg/μL) in PBS were immobilized on a NeutrAvidin (NLC) sensor chip (Bio-Rad) in vertical orientation at a flow rate of 30 μL/min.

Concentrations of 0.625, 1.25, 2.5, 5, and 10 μM H1-HA1 protein in PBS were run across the surface in horizontal orientation at a flow rate of 100 μL/min for 60 s with a dissociation time of 600 s. Data were analyzed with the ProteON Manager software, and binding constants were determined using a simple 1:1 Langmuir model. Equilibrium dissociation constants (K_D_) were calculated from association and dissociation rate constants (K_D_ = k_d_/k_a_).

### Western blot

Purified H1-HA1 protein was separated by sodium dodecyl sulfate polyacrylamide gel electrophoresis (SDS-PAGE) and electrophoretically transferred to a polyvinylidene fluoride membrane. The membrane was washed, blocked with 5% BSA in PBST at room temperature for 1 h, and incubated with 5′-biotinylated ssDNA aptamer in PBST (500 ng/mL) for 1 h. After four washes, the membrane was incubated with streptavidin-HRP for 1 h, washed again, and bands were visualized using an enhanced chemiluminescence (ECL) system and ImageQuant LAS 4000 (GE Healthcare Bio-Sciences, Piscataway, NJ). For specificity analysis, H5-HA1 and GST proteins were treated accordingly and served as controls.

### Circular dichroism (CD) spectroscopy and prediction of G-quadruplex structures

CD spectra were collected as previously described [28], using a Chirascan-plus CD spectrometer (Applied Photophysics, Leatherhead, Surrey, UK). Aptamers (25 μM) were resuspended in 10 mM Tris/HCl (pH 7.5) buffer containing 100 mM KCl. CD spectra data were obtained from 200~320 nm at a step size of 1 nm, a 0.2 s time-per-point, and a bandwidth of 1 nm. Each spectrum was an average of three scans at room temperature and was buffer baseline corrected. The presence of G-quadruplex structures in aptamers was predicted by quadruplex-forming G-rich sequences (QGRS) Mapper [29,30].

### Cell culture

Cells of the human embryonic kidney epithelial cell line HEK293T (ATCC, USA) were cultured in Dulbecco’s modified minimal essential medium supplemented with 100 U penicillin and 10% fetal bovine serum at 37°C and 5% CO_2_.

### Flow cytometry analysis

To confirm the selectivity of ssDNA aptamers, HEK293T cells were harvested with trypsin-EDTA, washed three times with 1 mL of PBS, added to a pre-incubated protein-aptamer complex of 100 μg GST-H1-HA1 protein and 8 μg FITC-labeled aptamer in PBS, and incubated at 4°C for 30 min. The cells were washed, blocked with 1% BSA in PBS for 30 min, incubated with a phycoerythrin (PE)-conjugated anti-GST antibody (Abcam, Cambridge, UK) in PBS at 4°C for 30 min, washed, fixed in 4% paraformaldehyde solution, and suspended in 500 μL PBS with 10% fetal calf serum and 1% NaN_3_. Fluorescence was determined with a Guava easyCyte flow cytometer (Millipore, Billerica, MA) by counting 10,000 events. GST-H5-HA1 was used as a negative control.

## Results

### Purification and amino acid sequence alignment of GST-tagged HA1 proteins

The HA1 genes were cloned in the pGEX-4T-1 expression vector (S1A Fig), transformed into Rosetta 2(DE3) cells, and GST-tagged proteins H1-HA1 and H5-HA1 were purified by glutathione-agarose affinity chromatography and Sephadex G-100 gel-filtration chromatography, as described in Supporting Information. Purified HA1 protein was identified as a band of the expected mass of ~64 kDa on 10% SDS-PAGE gels stained with Coomassie brilliant blue and on western blots using anti-GST antibody (S1B Fig). BLAST (Basic Local Alignment Search Tool) was used to compare the amino acid sequence similarity of H1-HA1 and H5-HA1. Since H1 and H5 belong to the same group (group 1) of hemagglutinins, their amino acid sequences were expected to be highly similar. The BLAST result showed that they have 55% identity and 73% similarity (S1C Fig). To determine whether the purified HA1 proteins have biological activity, hemagglutination assay was carried out with chicken red blood cells (RBCs). S2 Fig shows that both HA1 proteins were able to efficiently agglutinate the erythrocytes, and subsequent SELEX procedure was performed.

### In vitro selection of ssDNA aptamers specific for H1-HA1 rather than H5-HA1, and determination of binding affinity

**Fig 1:**
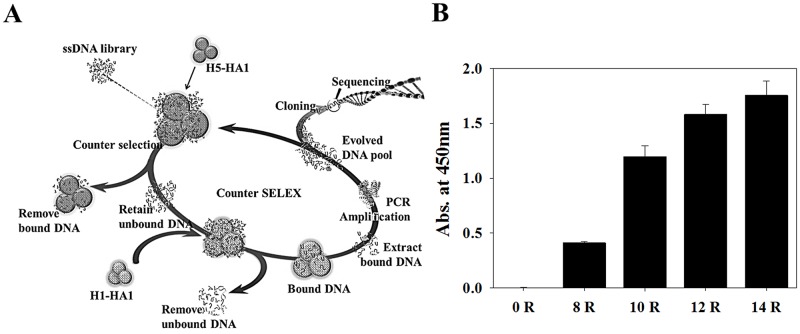
*In vitro* selection of ssDNA aptamers and specific binding activity of the ssDNA pool. (A) Schematic representation of the counter-SELEX procedure. After removing ssDNA species binding nonspecifically to glutathione agarose beads, the ssDNA pool was incubated with H5-HA1 (negative control) and centrifuged to remove H5-HA1-binding ssDNAs, after which the unbound ssDNAs were incubated with H1-HA1 (target protein), and the H1-HA1-bound DNAs were extracted using phenol-chloroform and amplified by PCR. After 14 rounds of selection, the enriched DNA was PCR-amplified, cloned, and sequenced. (B) Specific binding activity as measured by ELISA after 8, 10, 12, and 14 rounds of selection. GST-tagged H1-HA1 (100 nM) incubated on selected DNA-coated plates and analyzed using anti-GST antibody-HRP with TMB color detection.

**Fig 2:**
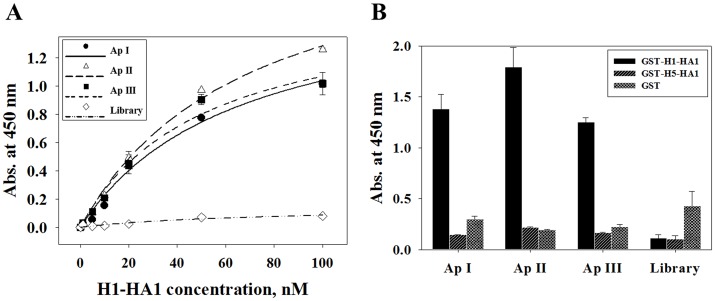
Binding analysis of selected ssDNA aptamers by ELISA. (A) Affinity measurements of selected ssDNA aptamers to H1-HA1 by ELISA. Immobilized biotinylated ssDNA aptamers were incubated with increasing concentrations of GST-H1-HA1, and binding was detected with anti-GST antibody-HRP. The biotinylated ssDNA library was used as negative control (◊). Graphs were fitted to the Michaelis-Menten equation, and K_d_ values were calculated as 64.76 ± 18.24 nM for ApI (●), 69.06 ± 12.34 nM for ApII (△), and 50.32 ± 14.07 nM for ApIII (■). (B) 100 nM GST-H1-HA1, GST-H5-HA1, or GST proteins incubated with biotinylated aptamers immobilized on streptavidin-coated plates to compare binding affinities.

To select specific ssDNA aptamers that can distinguish H1-HA1 from H5-HA1, counter-SELEX was performed with an ssDNA library of 88-mers containing a randomized sequence region of 45 nucleotides in the center, followed by lambda exonuclease digestion, as shown in Fig 1A. Enrichment of selected ssDNA aptamers specific for H1-HA1 protein was assessed by ELISA on the basis of the interaction between H1-HA1 protein and biotinylated ssDNA aptamers. A total of 14 cycles of selection were performed, and ssDNA pools isolated after rounds 8, 10, 12, and 14 were tested for binding affinity. As shown in Fig 1B, the absorbance at 450 nm increased significantly from round 10 onwards. After 14 cycles of selection, we obtained 22 independent ssDNA aptamers and measured their binding affinity by ELISA and SPR, as described in Materials and Methods. Binding curves were fitted to a Michaelis-Menten equation (Abs = A[H1-HA1]/(K_d_ + [H1-HA1], where Abs is the absorbance, A is the amplitude, [H1-HA1] is the concentration of H1-HA1, and K_d_ is the dissociation constant), and the amplitude and K_d_ value of each ssDNA aptamer were obtained. Out of 22 candidates, three aptamers, named ApI, ApII, and ApIII, were found to have high binding affinities to H1-HA1, but not to H5-HA1. The K_d_ values of ApI, ApII, and ApIII were 64.76 ± 18.24 nM, 69.06 ± 12.34 nM, and 50.32 ± 14.07 nM, respectively, as determined from the binding curve (Fig 2A). H5-HA1 and GST served as negative controls to which the selected aptamers did not bind (Fig 2B).

**Fig 3:**
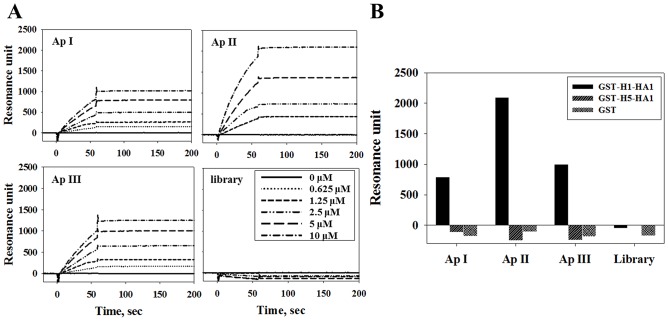
Affinity measurements of biotinylated ssDNA aptamers by SPR. (A) Biotinylated aptamers immobilized on an NLC sensor chip and interacting with various amounts of GST-H1-HA1. Equilibrium dissociation constants K_D_ were calculated from association and dissociation rate constants (K_D_ = k_d_/k_a_) as 96.6 nM for ApI, 1.09 μM for ApII, and 293 nM for ApIII. (B) Interaction of 10 μM H1-HA1, H5-HA1, or GST with aptamers immobilized on an NLC sensor chip, as measured by SPR.

Binding affinities of the three selected aptamers were also measured by SPR technology (Fig 3A). The K_D_ values were calculated from the resonance unit as 96.6 nM (ApI), 1.09 μM (ApII), and 293 nM (ApIII), whereas the selected aptamers did not bind to GST-H5-HA1 or GST used as negative controls (Fig 3B).

### Western blot analysis

**Fig 4:**
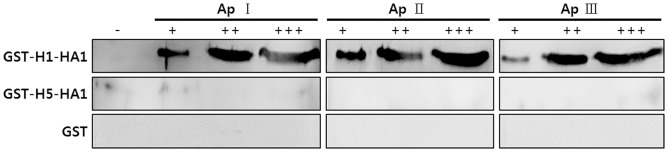
Western blot analysis using aptamers. Various amounts of GST-H1-HA1, GST-H5-HA1, and GST proteins were separated by SDS-PAGE, incubated with 5′-biotinylated aptamers, detected by streptavidin-HRP and ECL visualization (−, 0 μg; +, 1 μg; ++, 5 μg; +++, 10 μg).

Western blotting was performed to determine whether the selected ssDNA aptamers could be used as molecular probes for H1 protein recognition. Various amounts of H1-HA1 protein (0, 1, 5, and 10 μg) were analyzed by western blot using ssDNA aptamers (500 ng/mL). Fig 4 shows that the band intensities increased as the amount of H1-HA1 protein increased, indicating that the selected aptamers were bound to the H1-HA1 protein in a dose-dependent manner. Control experiments with GST-H5-HA1 and GST showed that the three aptamers did not bind to these proteins, corresponding to the results of ELISA and SPR, as described above. To rule out the non-specific binding of aptamers to cells, another western blot analysis was carried out using whole cell lysates. S3 Fig shows that non-specific binding was not detectable.

### Structural analysis of three selected aptamers

**Fig 5:**
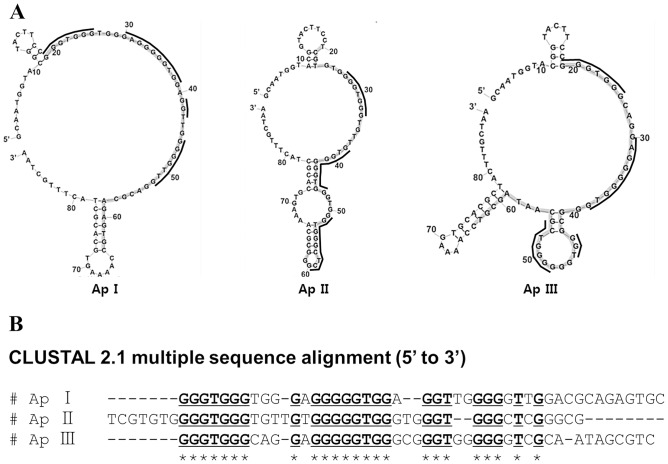
Predicted structures and sequence alignments of selected aptamers. (A) Mfold-predicted secondary structures of selected aptamers with shaded nucleotides representing the 45-nucleotide random sequence region. (B) Conserved sequences identified by ClustalW2 indicated by asterisks and underlines, and shown in (A) as solid lines.

**Fig 6:**
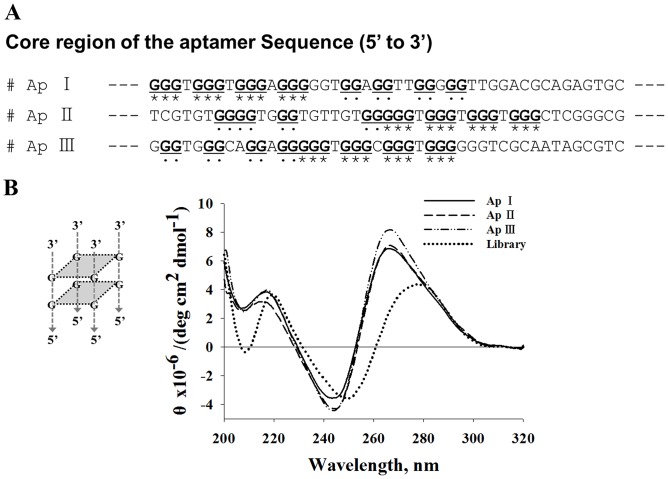
G-rich sequence of the core region and CD spectra of selected ssDNA aptamers. (A) G-rich sequences participating in the formation of G-quadruplex structures as predicted by QGRS Mapper in bold type and underlined. (B) CD spectra as collected on a Chirascan-plus CD spectrometer at 200–320 nm.

The secondary structure of the selected aptamers was predicted using Zuker’s Mfold program (Fig 5A), showing that the 45-nucleotide random sequence region (shaded) forms a major loop (ApI) or a major loop with a small hairpin (ApII and ApIII). Bioinformatic analysis using ClustalW2 (Fig 5B) revealed that the selected aptamers comprise of characteristic clusters of nucleotides in the 45-nucleotide random sequence region (indicated by asterisks) and contain seven nucleotides (GGGTGGG) followed by (G(N)GGGGGTGG), (GGT), and (GGG(N)T(N)G). We also found that the G-rich sequences were located in the large major loop (ApI, ApII, and ApIII) as well as in the hairpin regions (ApII and ApIII) (solid line in Fig 5A). Although the exact binding structure is still unclear, the G-rich sequence might be involved in the interaction with H1-HA1. Interestingly, we also found that each aptamer was likely to have two plausible G-quadruplex structures in the 45-nucleotide random sequence region. Therefore, we used QGRS Mapper (representing underlined dots and asterisks in Fig 6A) and circular dichroism to determine whether each aptamer has a G-quadruplex structure. In Fig 6B, a positive peak at 280 nm was obtained with the starting ssDNA library, indicating a normal DNA population [31]. On the other hand, the selected aptamers were found to have parallel G-quadruplex structures, which were identified by a positive maximum peak at 266 nm and a negative minimum peak at 244 nm [32].

### Flow cytometry analysis

**Fig 7:**
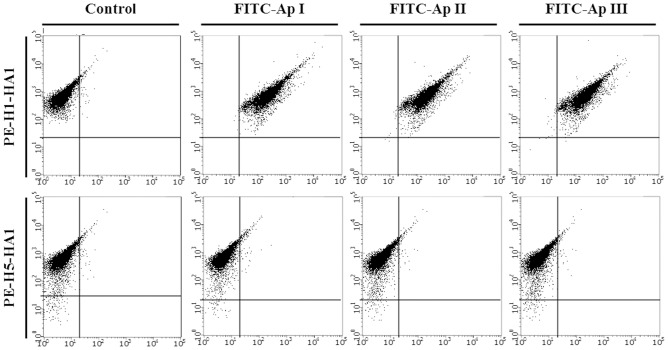
Flow cytometry analysis. HEK 293T cells incubated with aptamer-protein complexes containing GST-H1-HA1 or GST-H5-HA1 as analyzed by flow cytometry. The distributions of proteins (column) and selected aptamers (row) are shown.

To confirm that the selected aptamers were bound to the sialic acid-binding region of HA1, we performed a flow cytometry analysis (Fig 7). We pre-incubated FITC-labeled aptamers with H1-HA1 or H5-HA1 and added the complexes to HEK293T cells harboring sialic acid on the surface. As shown in Fig 7, the shifts of FITC and PE labels with H1-HA1 and H5-HA1 indicate that aptamer-H1-HA1 complexes were bound to sialic acid on the HEK293T cell surface, whereas H5-HA1 was bound to sialic acid with no aptamer attached. Therefore, the selected aptamers were found to bind selectively to H1-HA1 and not to H5-HA1, while they did not interfere with the sialic acid-binding ability of HA1 regardless of the subtype, suggesting that the aptamers were not binding to the sialic acid-binding region of HA1.

## Discussion

Upon influenza virus infection, surface glycoprotein HA recognizes and binds to sialic acid of the host cell membrane, leading to membrane fusion between the virus envelope and the endosome [33]. The identification of the type of HA present on the viral surface could aid in the determination of the species that can be infected and the type of sialic acid that is necessary (Siaα2-6Gal or Siaα2-3Gal) for viral infection [34]. Therefore, an efficient and accurate method to detect the several types of HA is important for the determination of the influenza subtype in infected species. When HA1 sequences of subtypes H1 and H5 were aligned by BLAST, the two proteins were found to have 55% identity and 73% similarity. Therefore, the development of an ssDNA aptamer that can distinguish between H1 and H5 was assumed to be useful for the subtle differentiation between two influenza subtypes. We constructed an ssDNA pool of 88-mers containing a randomized sequence region of 45 nucleotides in the center, which was screened using counter-SELEX in order to isolate ssDNA aptamers binding specifically to H1-HA1 protein, but not to H5-HA1. After 14 cycles of selection, we were able to select three ssDNA aptamers and measure their binding affinities to H1-HA1 by ELISA and SPR. Both methods produced similar results in a range of nanomolar K_d_ values. In addition to determining K_d_ values, ELISA and SPR experiments demonstrated that the fixed aptamers on the surface of a plate or chip were able to bind to the H1-HA1 protein. This indicates that appropriate protein-specific ssDNA aptamers would make good probes for the development of biosensors. Further analysis by western blotting demonstrated that the selected aptamers could be used as substitutes for commercial anti-H1-HA1 protein antibodies (S4 Fig). Based on the fact that aptamers are called “chemical antibodies” for their high affinity to target molecules, the isolation of good aptamers should be very attractive for the development of immunological reagents and diagnostic systems [35,36].

When we aligned the sequences of the selected aptamers using ClustalW2, we found that the aptamers have G-rich sequence clusters in the 45-random nucleotide region. Based on the fact that a characteristic G-rich sequence of nucleotides, (GG(N)_x_GG) or (GGG(N)_x_GGG), forms a G-quadruplex structure [37], we predicted those by QGRS Mapper [29,30], because G-rich sequences of the selected aptamers could fold into a G-quadruplex structure [38]. In addition to prediction, the structure of aptamers was analyzed by CD, showing that the selected aptamers have two G-quadruplex structures. According to a previous study, a stable G-quadruplex structure of selected aptamers has a strong binding capacity to a target protein [39]. Therefore, it is likely that a G-quadruplex structure plays an important role in the binding to H1-HA1, although the exact binding mode is still unclear [40].

In fact, there have been several attempts to discover nucleic acid aptamers against HA protein as a target. The Arnon group discovered a DNA aptamer that can prevent viral infection by blocking the receptor-binding region of HA (amino acid 91–261) regardless of the subtype (H1N1, H2N2, and H3N2) [41]. Using counter-SELEX, the Kumar group selected an RNA aptamer that can distinguish H3 subtypes (H3N2) of different strains [42], while they recently isolated an aptamer binding to H5N1 and H7N7 viruses and inhibiting HA-glycan interactions [43]. The Kim group isolated RNA aptamers that bind to H5-HA1 (H5N2) and suppress viral infection [44,45]. In most previous studies, the isolated aptamers were binding to the receptor-binding region of HA proteins. To determine whether our selected aptamers were also binding to the receptor-binding region of HA, thereby inhibiting the interaction with the receptor of the host cell, flow cytometry analysis was performed. Interestingly, our selected aptamers did not block the interaction between H1-HA1 protein and the receptor of the host cell, indicating that the aptamers bind to H1-HA1 in a region other than the receptor-binding region. Therefore, our selected aptamers are not expected to inhibit HA-mediated membrane fusion.

Considering the vital role of the HA protein in influenza infection, it is important to determine the HA subtype present in infected species for the purpose of diagnosis and prophylaxis. Therefore, methods for rapid and reliable detection of various subtypes of HA need to be established.

In the present study, we isolated new ssDNA aptamers that specifically bind to H1-HA1 protein and distinguish it from subtype H5-HA1. We anticipate that our selected aptamers can be used as sensitive probes for the development of new detection devices. In the future research, the selected aptamers will be modified to have functional groups for immobilization on the appropriate surface including gold nanoparticles or magnetic beads [46,47]. Combination of probe development with up-to-date chemical and physical methodology would expand the application of current aptamer-based system.

## Supporting Information

S1 Fig Purification and amino acid sequence alignment of H1-HA1 and H5-HA1. (A) Depiction of H1-HA1 and H5-HA1 expression vector. (B) Purification of GST-tagged H1-HA1 and H5-HA1. Lane M, molecular weight marker; lane 1, purified GST-H1-HA1; lane 2, western blot of purified GST-H1-HA1; lane 3, purified GST-H5-HA1; lane 4, western blot of purified GST-H5-HA1. The gel was stained with Coomassie brilliant blue. The western blot was performed using GST antibody-HRP. (C) Amino acid sequence alignment of H1-HA1 and H5-HA1 by BLAST. Asterisks and dots represent identities and positives, respectively.
(TIF)

S2 Fig Hemagglutination assay. The purified HA1 proteins have hemagglutination activity. When 30 μg of H1-HA1 was mixed with 40 μl of 1% (v/v) chicken RBCs, efficient agglutination of erythrocytes started. In case of H5-HA1, aggregation of erythrocytes started from 35 μg of the protein. Same experiments were repeated with BSA as a negative control.
(TIF)

S3 Fig Western blot analysis with whole cell lysates. The selected aptamers does not bind to cell lysates, which indicates no non-specific binding of aptamers to cell lysates. Beta-actin was used as a control.
(TIF)

S4 Fig Western blot analysis with commercial antibodies and the selected aptamers. The band intensities generated using commercial antibodies (HA1 and GST antibodies) and the selected aptamers were similar to each other.
(TIF)

S1 Methods Construction of expression vectors and purification of GST-tagged HA1. (DOCX)

S2 Methods Hemagglutination assay. (DOCX)

S3 Methods Western blot analysis with whole cell lysates. (DOCX)

S4 Methods Western blot analysis with commercial antibody. (DOCX)
