# Supplementary figures and images for: Isolation of Single-Stranded DNA Aptamers That Distinguish Influenza Virus Hemagglutinin Subtype H1 from H5
Source: PLoS One. 2015 Apr 22;10(4):e0125060. doi: 10.1371/journal.pone.0125060 (PMC4406500; doi:10.1371/journal.pone.0125060)

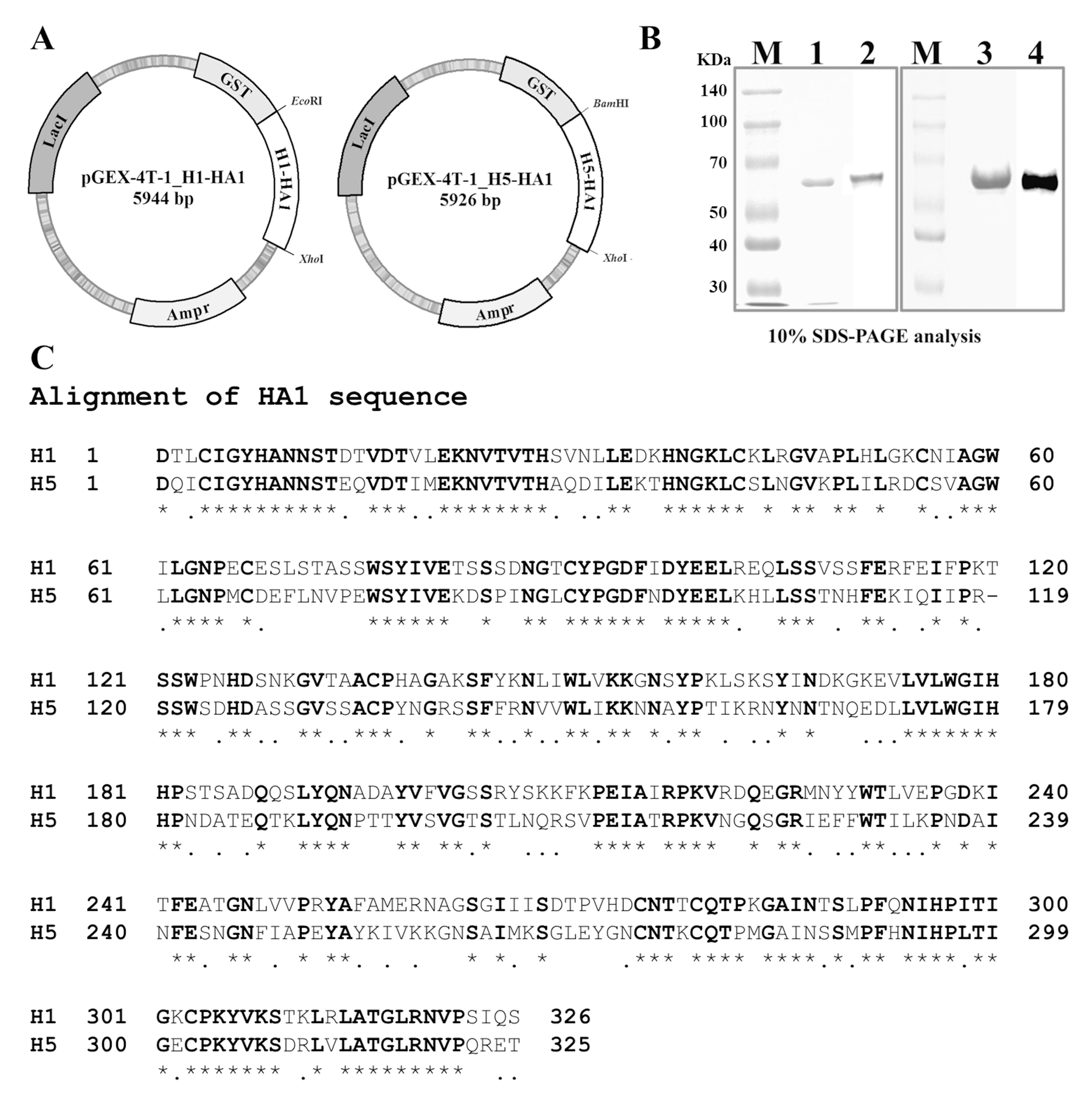

Supplement: S1 Fig — (A) Depiction of H1-HA1 and H5-HA1 expression vector. (B) Purification of GST-tagged H1-HA1 and H5-HA1. Lane M, molecular weight marker; lane 1, purified GST-H1-HA1; lane 2, western blot of purified GST-H1-HA1; lane 3, purified GST-H5-HA1; lane 4, western blot of purified GST-H5-HA1. The gel was stained with Coomassie brilliant blue. The western blot was performed using GST antibody-HRP. (C) Amino acid sequence alignment of H1-HA1 and H5-HA1 by BLAST. Asterisks and dots represent identities and positives, respectively. (TIF) [file pone.0125060.s001.tif]

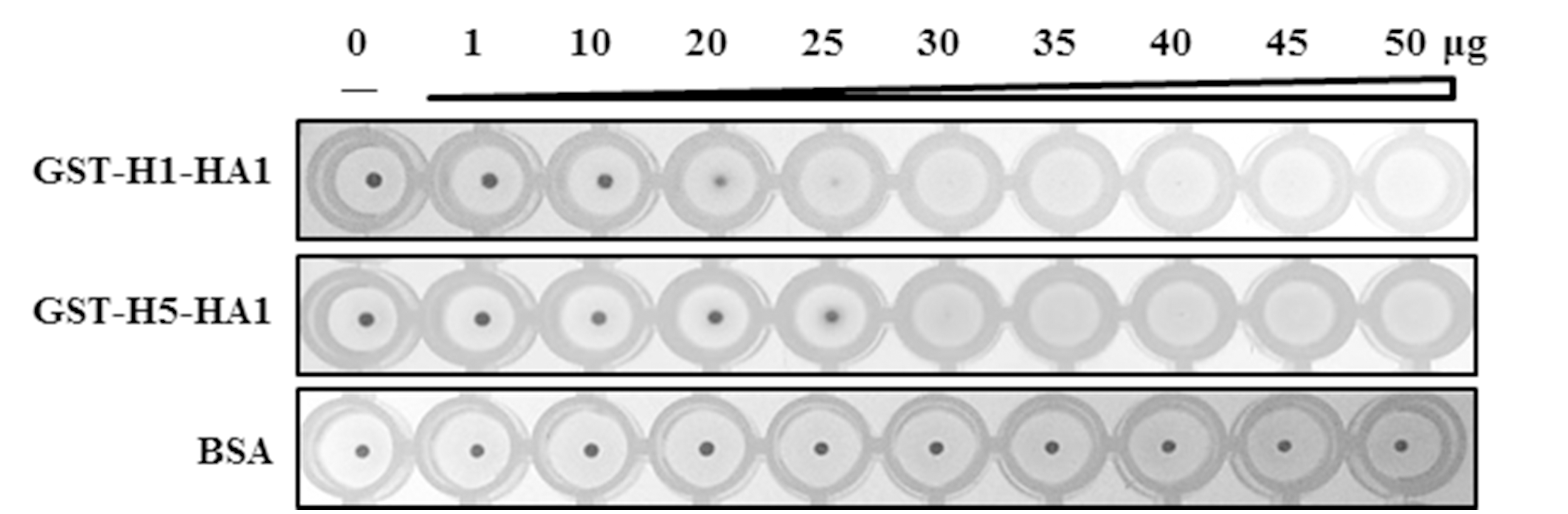

Supplement: S2 Fig — The purified HA1 proteins have hemagglutination activity. When 30 μg of H1-HA1 was mixed with 40 μl of 1% (v/v) chicken RBCs, efficient agglutination of erythrocytes started. In case of H5-HA1, aggregation of erythrocytes started from 35 μg of the protein. Same experiments were repeated with BSA as a negative control. (TIF) [file pone.0125060.s002.tif]

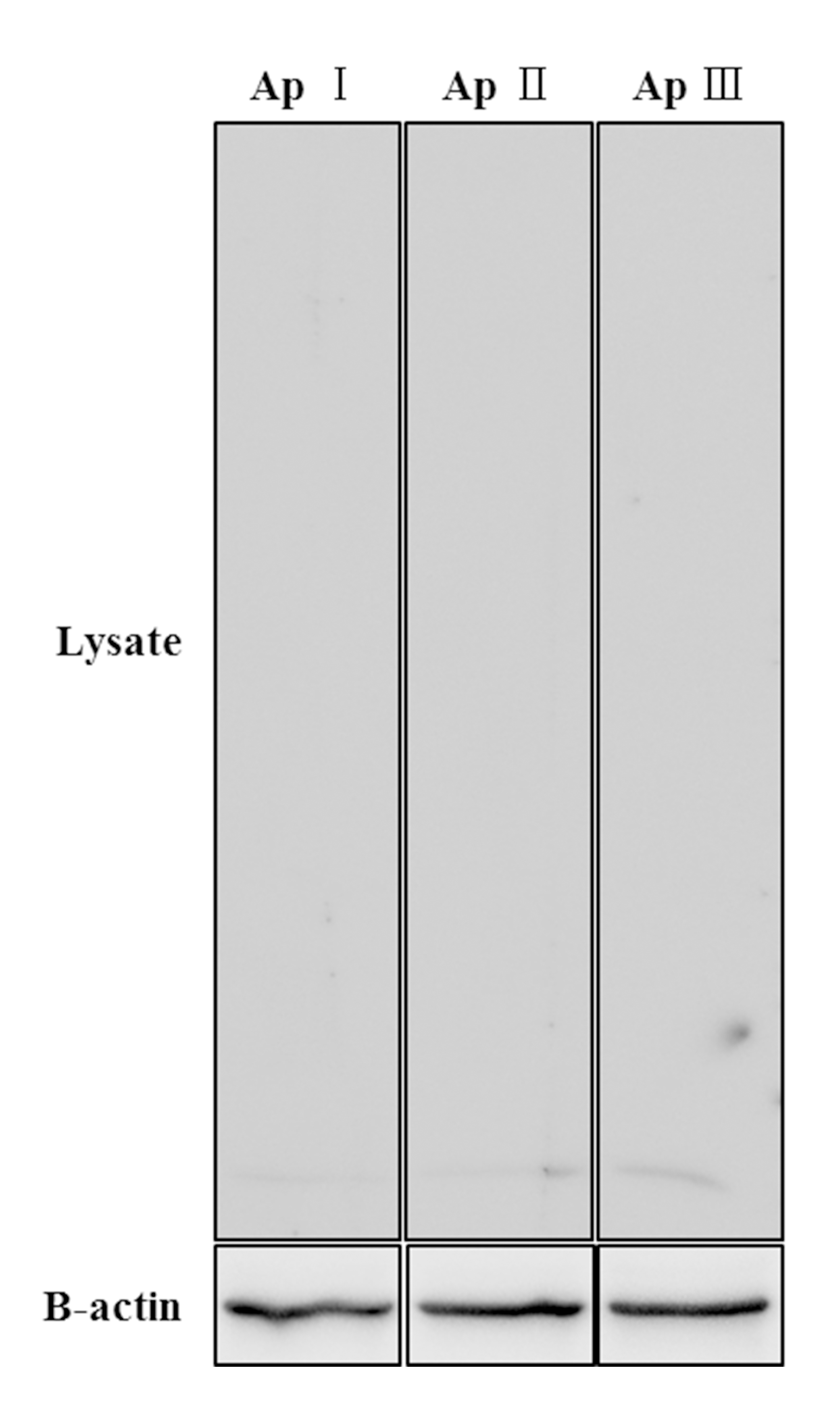

Supplement: S3 Fig — The selected aptamers does not bind to cell lysates, which indicates no non-specific binding of aptamers to cell lysates. Beta-actin was used as a control. (TIF) [file pone.0125060.s003.tif]

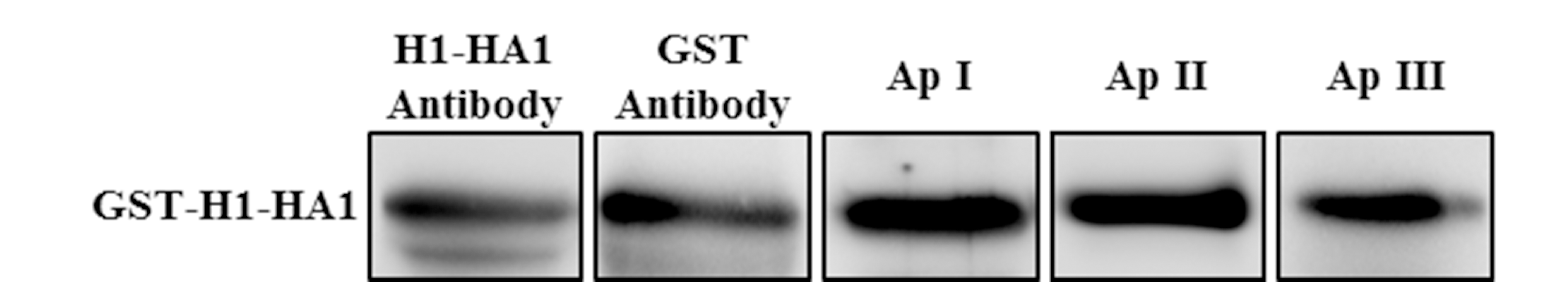

Supplement: S4 Fig — The band intensities generated using commercial antibodies (HA1 and GST antibodies) and the selected aptamers were similar to each other. (TIF) [file pone.0125060.s004.tif]
