# Supplementary material for: Isolation of Single-Stranded DNA Aptamers That Distinguish Influenza Virus Hemagglutinin Subtype H1 from H5
Source: PLoS One. 2015 Apr 22;10(4):e0125060. doi: 10.1371/journal.pone.0125060 (PMC4406500; doi:10.1371/journal.pone.0125060)
Supplement: S1 Methods — (DOCX) [file pone.0125060.s005.docx]

***Construction of expression vectors***

Genomic RNA of A/Korea/01/2009 (H1N1, Genbank ID : GQ131023.1) was provided by the Korea Centers for Disease Control and Prevention (KCDC). Complementary DNA (cDNA) was obtained by reverse transcription using a High Capacity RNA-to-cDNA Kit (Life Technologies, Carlsbad, CA), and the receptor-binding domain of the hemagglutinin (H1-HA1) gene was amplified by PCR, using a G-Taq DNA polymerase (Cosmogenetech, Seoul, Korea) with an *EcoRI* forward primer (5′-GTGGTGGAATTCGACACATTATGTATAGGTTATCATGCG-3′) and an *XhoI* reverse primer (5′-CATATTCTCGAGTCATCTAGATTGAATAGACGG-3′) (IDT). The PCR product was gel-purified, digested with *EcoRI/XhoI*, and ligated with an equally digested pGEX-4T-1 vector to construct the plasmid pGEX-4T-1/H1-HA1 (S1A Fig.). An expression vector encoding the hemagglutinin HA1 domain of subtype H5 influenza virus strain A/duck/Korea/GJ54/2004 (H5N2, Genbank ID : GU351859.1), pGEX-4T-1/H5-HA1, was constructed accordingly, using *BamHI/XhoI* for digestion and ligation.

***Purification of GST-tagged HA1***

GST-tagged H1-HA1 protein was expressed in *Escherichia coli* Rosetta 2(DE3)^TM^ cells (Millipore). Transformed cells were grown at 37°C to an OD_600_ of 0.5~0.6 and further incubated at 4°C for 3 h. Isopropyl β-D-thiogalactopyranoside (IPTG, 0.5 mM) was added, and the cells were incubated at 37°C with shaking for 4 h. After harvest by centrifugation, the cell pellets were resuspended in lysis buffer (0.3 M NaCl, 1 mM dithiothreitol (DTT), and 1% Triton X-100 in PBS), lysed by sonication, and centrifuged at 13,000 ×*g* for 1 h. The resulting supernatant was affinity-purified using a glutathione agarose bead column. The column was washed with wash buffer 1 (1 mM DTT and 1% Triton X-100 in PBS), wash buffer 2 (50 mM Tris/HCl; pH 8.0 and 1 mM DTT), and protein was eluted with buffer 3 (50 mM Tris/HCl; pH 8.0, 1 mM DTT, 0.1% Triton X-100, and 10 mM glutathione). The purest samples were combined and ultrafiltrated using an Amicon stirred cell with YM-30 (Millipore). The H1-HA1 protein was further purified by gel filtration chromatography. Samples were applied to a Sephadex G-100 (Sigma-Aldrich, St. Louis, MO, USA) column, and protein was eluted using buffer 4 (25 mM Tris/HCl; pH 7.5 and 0.3 M NaCl) at a flow rate of 0.3 mL/min. Eluted fractions were collected and analyzed by 10% SDS-PAGE (S1B Fig.). Protein concentration was determined by Bradford assay, and the samples were stored at −80°C. For bioinformatic analysis, BLAST (Basic Local Alignment Search Tool) was used to compare the similarities of H1-HA1 and H5-HA1 amino acid sequences (S1C Fig.).
