# Supplementary material for: Isolation of Single-Stranded DNA Aptamers That Distinguish Influenza Virus Hemagglutinin Subtype H1 from H5
Source: PLoS One. 2015 Apr 22;10(4):e0125060. doi: 10.1371/journal.pone.0125060 (PMC4406500; doi:10.1371/journal.pone.0125060)
Supplement: S2 Methods — (DOCX) [file pone.0125060.s006.docx]

***Hemagglutination assay***

To determine whether GST-HA1 proteins used in this study have the biological activity, we performed hemagglutination assay. The assay is based on the ability of the HA1 proteins to bind to and agglutinate avian and mammalian erythrocytes. Various amounts of HA1 proteins (50 μg to 1 μg) in PBS were placed in a 96-well plate. Then 40 μl of 1% (v/v) chicken red blood cells (RBCs) in PBS was added to the each well. The reaction mixture was incubated for 1 h at room temperature, after which the plate was photographed (S2 Fig.).
