# Supplementary material for: Isolation of Single-Stranded DNA Aptamers That Distinguish Influenza Virus Hemagglutinin Subtype H1 from H5
Source: PLoS One. 2015 Apr 22;10(4):e0125060. doi: 10.1371/journal.pone.0125060 (PMC4406500; doi:10.1371/journal.pone.0125060)
Supplement: S3 Methods — (DOCX) [file pone.0125060.s007.docx]

***Western blot analysis with whole cell lysates***

To rule out non-specific binding of the selected aptamers to off-target cellular proteins, we performed western blot analysis using whole cell lysates. HEK293T cells were lysed in NP40 cell lysis buffer (Invitrogen, Carlsbad, CA) by sonication. Then, 5 μg of lysates was separated by SDS-PAGE and further western blot analysis with the selected aptamers was performed (S3 Fig.) as described in ‘Materials and Methods’.
