# Supplementary material for: Isolation of Single-Stranded DNA Aptamers That Distinguish Influenza Virus Hemagglutinin Subtype H1 from H5
Source: PLoS One. 2015 Apr 22;10(4):e0125060. doi: 10.1371/journal.pone.0125060 (PMC4406500; doi:10.1371/journal.pone.0125060)
Supplement: S4 Methods — (DOCX) [file pone.0125060.s008.docx]

***Western blot analysis with commercial antibody***

Western blot analysis was performed to determine if the selected aptamers can be used as a substitute for commercial HA1 antibody. 5 μg of purified GST-tagged H1-HA1 protein was separated by SDS-PAGE and transferred to a PVDF membrane. The membranes were blocked with 5% BSA in PBST at room temperature for 1 h, and then incubated with 500 ng 5′-biotinylated selected aptamers in PBST for 1 h. After 4 washes, the membranes were incubated with streptavidin-HRP (Pierce Biotechnology, Rockford, IL) for 1 h. After washing, the membranes were visualized by enhanced chemiluminescence (ECL) reaction and exposed to LAS 4000 (GE Healthcare). The same experiments were repeated using 500 ng GST antibody (Z-5)-HRP (Santa Cruz Biotechnology, Dallas, TX) or 500 ng anti-HA1 (H1N1) antibody (Abcam, Cambridge, MA) instead of the selected ssDNA aptamer to compare the efficacy of the selected aptamer with commercial antibodies. After the blotting, blocking, and washing steps, the membranes were incubated with GST antibody (Z-5)-HRP or anti-HA1 (H1N1) antibody. In the case of incubation with GST antibody (Z-5)-HRP, membranes were visualized by the ECL reaction without further treatment. When the membrane was incubated with anti-HA1 (H1N1) antibody, the membrane was also reacted with the goat anti-rabbit IgG-HRP (Santa Cruz Biotechnology, Dallas, TX) prior to the ECL reaction step. S4 Fig. shows that the ssDNA aptamer isolated in this study could be used as an alternative to the antibody for the detection of H1-HA1 protein.
